# Pupillary Light Reflexes in Severe Photoreceptor Blindness Isolate the Melanopic Component of Intrinsically Photosensitive Retinal Ganglion Cells

**DOI:** 10.1167/iovs.17-21909

**Published:** 2017-07

**Authors:** Jason Charng, Samuel G. Jacobson, Elise Heon, Alejandro J. Roman, David B. McGuigan, Rebecca Sheplock, Mychajlo S. Kosyk, Malgorzata Swider, Artur V. Cideciyan

**Affiliations:** 1 Scheie Eye Institute, Perelman School of Medicine, University of Pennsylvania, Philadelphia, Pennsylvania, United States; 2 Department of Ophthalmology and Vision Sciences, The Hospital for Sick Children, University of Toronto, Toronto, Ontario, Canada

**Keywords:** *AIPL1*, Batten disease, *CEP290*, *CLN3*, *GUCY2D*, *IQCB1*, Leber congenital amaurosis, LCA1, LCA10, melanopsin, *NPHP5*, *NPHP6*, *RPGRIP1*

## Abstract

**Purpose:**

Pupillary light reflex (PLR) is driven by outer retinal photoreceptors and by melanopsin-expressing intrinsically photosensitive retinal ganglion cells of the inner retina. To isolate the melanopic component, we studied patients with severe vision loss due to Leber congenital amaurosis (LCA) caused by gene mutations acting on the outer retina**.**

**Methods:**

Direct PLR was recorded in LCA patients (*n* = 21) with known molecular causation and severe vision loss. Standard stimuli (2.5 log scot-cd.m^−2^; ∼13 log quanta.cm^−2^.s^−1^; achromatic full-field) with 0.1- or 5-second duration were used in all patients. Additional recordings were performed with higher luminance (3.9 log scot-cd.m^−2^) in a subset of patients.

**Results:**

The LCA patients showed no detectable PLR to the standard stimulus with short duration. With longer-duration stimuli, a PLR was detectable in the majority (18/21) of patients. The latency of the PLR was 2.8 ± 1.3 seconds, whereas normal latency was 0.19 ± 0.02 seconds. Peak contraction amplitude in patients was 1.1 ± 0.9 mm at 6.2 ± 2.3 seconds, considerably different from normal amplitude of 4.2 ± 0.4 mm at 3.0 ± 0.4 seconds. Recordings with higher luminance demonstrated that PLRs in severe LCA could also be evoked with short-duration stimuli.

**Conclusions:**

The PLR in severe LCA patients likely represents the activation of the melanopic circuit in isolation from rod and cone input. Knowledge of the properties of the human melanopic PLR allows not only comparison to those in animal models but also serves to define the fidelity of postretinal transmission in clinical trials targeting patients with no outer retinal function.

Steady-state pupil size and its dynamic changes are strongly influenced by the ambient light and previous light history through the pupillary light reflex (PLR).^1,2^ Depending on the temporal and spectral properties of the light and ocular adaptation conditions, the normal PLR is driven by melanopsin-containing intrinsically photosensitive retinal ganglion cells (ipRGCs),^3,4^ which constitute a small proportion of all RGCs,^5^ as well as rod and cone photoreceptors of the outer retina.^6,7^ Since rods are by far the most sensitive among all of the PLR photoreceptors, rod-dominated PLRs can be recorded in dark-adapted normal human eyes illuminated homogeneously across the visual field with lights that are above rod threshold but below cone and ipRGC thresholds.^8^ With high light levels, however, all ocular photoreceptors are activated, and signals from outer and inner retinal photoreceptors combine in a complex manner to drive the PLR.^4^ Isolation of the melanopic PLR^9^ in vivo without interference from outer retinal photoreceptors has been achieved in mice where phototransduction of rods and cones has been genetically abolished.^7^ In non-human primates, on the other hand, melanopic PLR has been isolated with the use of toxins that block transmission of signaling from photoreceptors to postreceptoral neurons.^10^ Normal human melanopic PLR component has been estimated with the use of special stimulus and adaptation conditions.^4,11–15^ Alternatively, the postillumination pupil response (PIPR) has been used to evaluate the loss of melanopic PLR in glaucoma.^16–20^

Another approach to isolating human melanopic PLR is to consider patients with severe rod and cone photoreceptor dysfunction but retained inner retinal structure, similar to the genetically engineered mice lacking outer retinal photoreception. To our knowledge, the literature includes PLRs recorded in four such patients.^8,21–23^ In one case,^21^ temporal profile and amplitude of the recorded PLR was not provided, but the sensitivity spectrum was suggestive of melanopsin absorption. The other three cases^8,22,23^ showed slow and small contractions to a bright blue stimulus presented in the dark, suggestive of activation of melanopic pathways. Here we examine the properties of PLR from a cohort of rare patients with different molecular forms of Leber congenital amaurosis (LCA) and severe retina-wide loss of rod- and cone-mediated visual function from early life to better understand the properties of the human melanopic PLR.

## Methods

### Human Subjects

Patients with different genetic forms of severe LCA (*n* = 21; Supplementary Table S1) with no form vision or motion perception (visual acuities of light perception [LP] and no light perception [NLP]) and subjects with healthy vision (*n* = 4; average 33.2 years; range, 23–54 years) participated in this study. Retinal structure and function phenotype from most of the patients have been published previously.^24–32^ Excluded were data from six patients (three with *CEP290* and one each with *NPHP5*, *RPGRIP1*, and *GUCY2D* mutations) with LP vision but detectable transient PLRs to our standard short stimulus, as well as two patients (one *AIPL1* and one *GUCY2D*) in whom visibility of pupils during the stimulus was obstructed. All subjects were treated in accordance with the tenets of the Declaration of Helsinki, and informed consents were obtained from all patients. The research was approved by the institutional review board at the University of Pennsylvania.

### Pupillometry

Two pupillometers were utilized in this study. Pupillometer I was the “standard” equipment reported previously.^27,28,31–37^ In brief, white full-field stimuli of short (0.1 second) or long (5 seconds) duration were used with a maximum luminance of 2.5 log scot-cd.m^−2^ (2.4 log phot-cd.m^−2^) presented monocularly in the dark-adapted (>1 hour) state. For each stimulus presented, video clips were digitized (PMR-202, iRecord Pro+; Streaming Networks, Inc., Santa Clara, CA, USA) from ∼5 seconds before stimulus onset up to ∼30 seconds after stimulus offset. The pupil was imaged with an infrared-sensitive video camera (LCL-903HS; Watec America Corp., Las Vegas, NV, USA) and a macro zoom lens (MLH-10X; CBC, Ltd., Tokyo, Japan). The focus and magnification were optimized before the first recording, and a 6-mm-diameter calibration target was imaged after the last recording to allow determination of absolute pupil size.

In a subset of four of the patients (P7, P10, P11, P14), additional recordings were performed with a pupillometer II (Roland Consult Pupillometer, Brandenburg a.d. Havel, Germany) with a maximum luminance of 3.9 log scot-cd.m^−2^ (3.6 log phot-cd.m^−2^). Shorter- (0.1 second) and longer- (1 second) duration stimuli were used. Videos clips were acquired at 30 frames per second starting from 1 second before the stimulus onset and lasting to 9 seconds after. Pupil magnification was fixed and calibrated.

Video clips from both pupillometers were analyzed frame-by-frame to define pupil boundaries by manually fitting an ellipse to the visible portion of the iris; the major axis of the ellipse was taken as the pupil diameter. Since LCA eyes with severe vision loss typically lack oculomotor control to retain stable primary gaze over time, we estimated the errors introduced by measuring the maximum pupil diameter at extremes of gaze (Supplementary Fig. S1). These errors are likely to be smaller than 5% of the pupil diameter at central gaze as long as >50% of the iris is visible at eccentric gaze. PLR parameters included amplitude defined as the baseline pupil diameter minus pupil diameter measured at fixed times after the stimulus onset; latency corresponding to the time amplitude reached the criterion value of 0.3 mm.^27,33,36^ Summary data are presented throughout as average ± 1 SD.

Full-field stimuli used in the current work are defined in terms of their scotopic and photopic luminance because of the common use of these units in human dark-adapted visual sensitivity literature. Spectral similarity between rod and ipRGC sensitivity spectra allow quick estimates of the efficacy of stimuli specified in terms of scotopic luminance in stimulating rods and ipRGCs. In addition, we estimated the radiant flux generated by our PLR stimuli at the human retina under simplifying assumptions. For pupillometer I, the estimate was performed in two steps. First we used the maximal green stimulus^33^ peaking near 520 nm (not otherwise used in the current manuscript) corresponding to a retinal luminance of 4 log_10_ scot-Td for an 8-mm-diameter pupil. The maximum radiant flux available at the cornea could be estimated as 9.7 log_10_ quanta.deg^−2^.s^−1^ by using the simplifying approximation of considering our narrow band stimulus as monochromatic (see Equation 8(2.4.4) in Wyszecki and Stiles^38^). For a human, that corneal radiant flux translates to 12.8 log_10_ quanta.cm^−2^.s^−1^ at the retina. The maximal white stimulus of pupillometer I (Supplementary Fig. S2A) would be expected to result in radiant flux approximately 0.2 log units higher to ∼13 log_10_ quanta.cm^−2^.s^−1^. For pupillometer II, the “white” stimulus (Supplementary Fig. S2B) corresponded to a retinal luminance of 5.6 log_10_ scot-Td. Using similar approximations, the radiant flux on the human retina was estimated to be 14.4 log_10_ quanta.cm^−2^.s^−1^. Thus the maximum stimuli available were approximately 2 log units (pupillometer I) and approximately 3 log units (pupillometer II) above ipRGC thresholds previously estimated to be ∼11 log quanta.cm^−2^.s^−1^ at 480 nm for human eyes.^4^ Our range was also similar to previous experimental recordings^7,39^ performed over 11–14 log quanta.cm^−2^.s^−1^.

### Full-Field Stimulus Testing

Full-field stimulus testing (FST) was performed in most (19/21) LCA patients to estimate the light sensitivity of the eyes as previously described.^40,41^ Briefly, a range of blue full-field stimuli (200 ms) were presented to dark-adapted eyes via a computer-driven simulator (Colordome; Diagnosys LLC, Littleton, MA, USA) and patients responded when they perceived the lights presented. FST loss for each patient was defined as the difference from the mean normal value for the same blue stimulus.

## Results

### Severe LCA Patients Lack a Transient PLR to Bright Light of Short Duration

**Figure 1:**
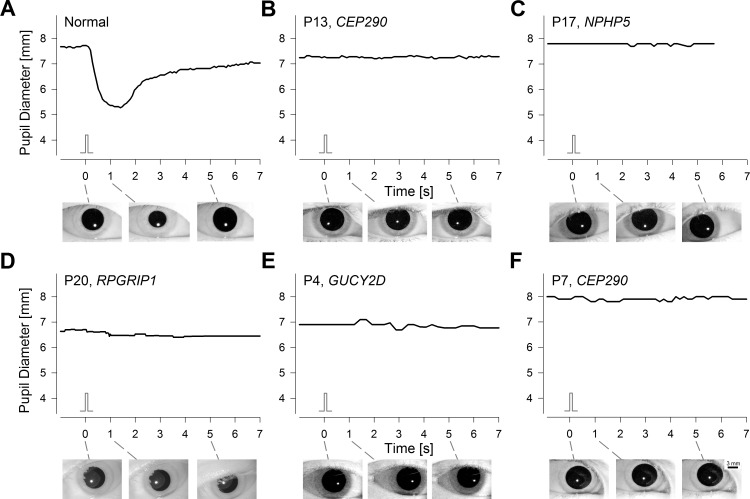
LCA patients with severe loss of light perception lack a transient PLR to a bright short-duration stimulus. (**A**) Representative normal response shows a fast pupillary constriction with a latency of ∼0.3 seconds and peak amplitude of 2.3 mm at ∼1 second. (**B**–**F**) Representative patients with LCA caused by *CEP290, NPHP5, RPGRIP1*, or *GUCY2D* mutations show no detectable changes in pupil diameter after the stimulus. Visual acuity of LCA patients ranged from LP to NLP. Stimulus monitors are shown. Iris images illustrate pupil diameter immediately before and 1 and 5 seconds after the stimulus for all subjects. Recordings were performed with pupillometer I using a bright (2.5 log scot-cd.m^–2^; 2.4 log phot-cd.m^–2^) short-duration (0.1 second) achromatic full-field stimulus presented in the dark to dark-adapted eyes.

LCA is considered the most severe form of inherited retinal degeneration with congenital abnormality of visual function. However, even within LCA there is a wide range of severity. Many LCA patients retain some vision originating from rod and/or cone photoreceptors. Not unexpectedly, these patients tend to have a transient PLR that is similar to a normal response in terms of latency, velocity, and amplitude, especially when adjusted for loss of light sensitivity of the underlying outer retinal photoreceptors.^8,23,27,28,31–36^ Patients included in the current work (Supplementary Table S1) represent the most severe end of the LCA spectrum with barely detectable or undetectable light pereceptions, and they tend to show no detectable PLR to the standard short and bright stimulus in the dark.^27,28,31–33^ Pupil diameter recordings from representatives of such LCA patients include P13 (a 24-year-old with NLP due to *CEP290* mutations; Fig. 1B), P17 (a 15-year-old with LP due to *NPHP5* mutations; Fig. 1C), P20 (a 14-year-old with LP due to *RPGRIP1* mutations; Fig. 1D), P4 (a 14-year-old with LP due to *GUCY2D* mutations; Fig. 1E), and P7 (a 13-year-old with LP due to *CEP290* mutations; Fig. 1F). In all cases, there was no detectable PLR in response to the standard stimulus as compared to the brisk large-amplitude transient PLR recordable in normal eyes (Fig. 1A). Maximal green or orange stimuli also resulted in no PLR (data not shown). Importantly, LCA patients without light perception tend to have unstable “wandering” eyes, which can complicate stable and continuous PLR recordings through baseline, stimulus, and poststimulus periods. In patients included, however, such methodological complexities were not applicable as demonstrated by clear visualization of the iris at key time points (Fig. 1, insets below).

Outer retinal rods and cones dominate the transient PLR evoked with short-duration stimuli.^10,33,42,43^ Thus, it is not unexpected for severe LCA patients to lack a transient PLR mediated by outer retinal photoreceptors. But why were there no detectable PLRs driven by melanopsin-containing ipRGCs? We hypothesized that the short duration of our standard stimuli, combined with the temporal tuning properties of the melanopic system,^3,44^ resulted in recording conditions below the melanopic PLR threshold.

### Long-Duration Stimuli Uncover a Slow PLR

**Figure 2:**
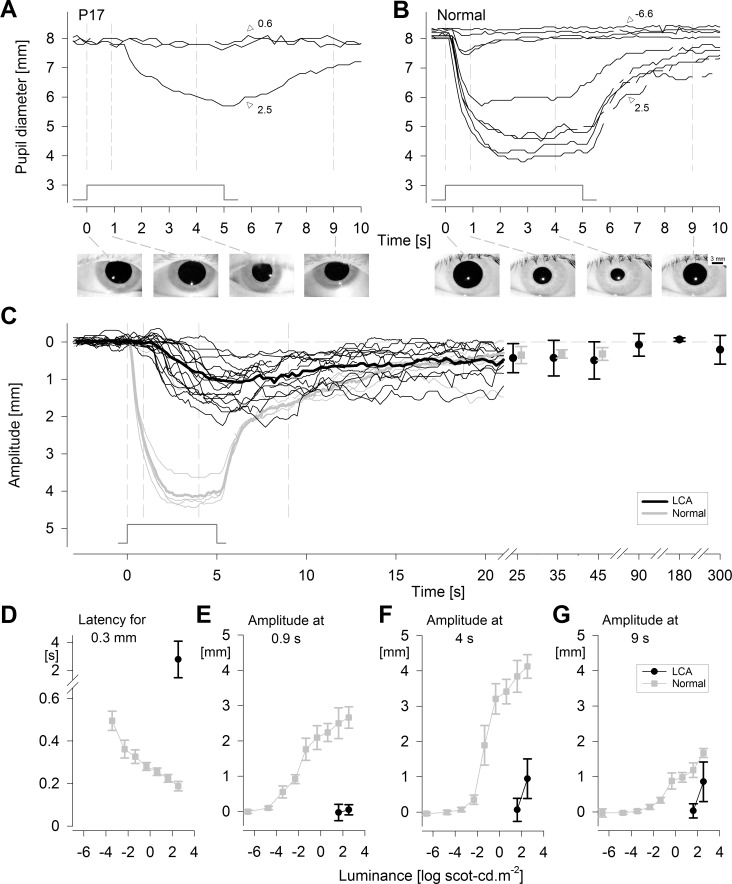
Slow and insensitive PLR exposed in severe LCA. (**A**) Changes in pupillary diameter in a representative LCA patient as a function of increasing luminance to 5-second-long stimuli. There was detectable PLR at the maximal luminance (2.5 log scot-cd.m^−2^; 2.4 log phot-cd.m^−2^) but no pupil constrictions at lower luminance (0.6 log scot-cd.m^−2^; 1.6 log scot-cd.m^−2^). (**B**) Changes in pupillary diameter in a representative normal eye as a function of increasing luminance. Normal threshold pupillary constriction is more than 7 log units below that of the LCA subject. Iris images demonstrate pupil diameter immediately before and 0.9, 4, and 9 seconds after stimulus for both subjects recorded with pupillometer I. (**C**) Pupil constriction amplitude as a function of time after stimulus onset in 18 individual LCA patients (*thin black traces*) compared to individual normal results (*thin gray traces*) at the maximal luminance. In a subset of patients (*black filled circles*) and in all healthy patients (*gray filled squares*), pupil recovery was able to be tracked at later time points. Baseline is represented by the *horizontal dashed line*. (**A**–**C**) Thin vertical *gray line*s demarcate stimulus onset and three time points quantified in panels **E**–**G**. Stimulus monitor shown. (**D**–**G**) Latency and amplitude parameters of the PLR in LCA (*black filled circles*) compared to mean normal (*gray filled squares*) as a function of luminance. *Error bars*: ±1 SD.

In order to evoke detectable PLRs, we used a longer-duration stimulus to take advantage of longer integration time of ipRGCs^3,44^ in these patients apparently lacking all outer retinal function. The consequences of the use of longer-duration stimuli are demonstrated (Fig. 2A) in a representative LCA patient (P17) lacking a detectable PLR to a short-duration stimulus of the same maximum luminance (Fig. 1C). Specifically, with the 5-second-long stimulus, there appears to be a steep sensitivity curve defined by no discernible pupil contractions to 0.6 and 1.6 log scot-cd.m^−2^ and a distinct PLR at 2.5 log scot-cd.m^−2^ with a slow latency (∼1.5 seconds to reach 0.3-mm amplitude) and small amplitude (∼2 mm). The PLR in the LCA patient differed considerably from that of a normal subject recorded with long-duration stimuli over a 9-log unit range (Fig. 2B). PLR threshold in the patient was approximately 7 log units elevated compared to normal. Importantly, PLR shape of the patient did not appear to match normal PLR evoked at any luminance, including those near threshold. There was clear visibility of the pupil throughout the recordings (Figs. 2A, 2B; insets below).

The majority (18/21 = 86%) of the LCA patients lacking a transient PLR to a short-duration stimulus demonstrated a detectable PLR to a longer-duration stimulus of the same luminance. There was some variability of the PLRs among individual LCA patients (Fig. 2C, black thin lines), but there was no overlap with normal results (Fig. 2C, thin gray lines) during the first 5 seconds after stimulus onset. On average, the normal PLR showed fast constriction following stimulus onset, plateauing at approximately 2.5 seconds. This was in stark contrast to the average PLR in LCA, with a delayed (∼2 seconds) latency and a slow constriction velocity. Upon stimulus offset, the normal PLR showed an accelerated initial redilation followed by a slower redilation. PLR in LCA had only a slow redilation phase. Normal and LCA PLRs became indistinguishable beyond 15 seconds (Fig. 2C).

To better understand the properties of the PLR evoked in LCA with severe vision loss, parameters were compared to normal results (Figs. 2D–G). The latency to reach a 0.3-mm criterion PLR amplitude in LCA ranged from 0.7 to 5.8 (mean ± SD; 2.8 ± 1.3) seconds, and this was significantly different from normal at the maximal luminance (0.2 ± 0.02 seconds; *P* = 0.001), at threshold (0.5 ± 0.05 seconds; *P* = 0.003), or any luminance in between (Fig. 2D). There was no overlap in latency between normal and LCA eyes (Figs. 2C, 2D). In normal subjects, the PLR amplitude measured at 0.9 seconds steadily grew with luminance, but in severe LCA, PLR was mostly not detectable at this time point (Fig. 2E). When measured at 4 seconds, on the other hand, the LCA patients showed detectable amplitudes at the maximal luminance (Fig. 2F) ranging from 0.1 to 1.8 (1.0 ± 0.6) mm. LCA amplitudes were significantly smaller than normal at the maximal luminance (4.1 ± 0.3 mm; *P* = 0.001) as well as normal PLRs evoked across most of the luminance range, except for those recorded with ∼4 log unit dimmer stimuli (Fig. 2F). The PLR amplitudes at 9 seconds in the patients (0.9 ± 0.6 mm) approached normal results (1.7 ± 0.13 mm) at maximal luminance (Fig. 2G); however they remained statistically smaller (*P* = 0.0001). Importantly, PLR amplitudes were undetectable with 1.6 log scot-cd.m^−2^ stimuli in all the patients (Figs. 2E–G) thus implying a response threshold occurring between 1.6 and 2.5 log scot-cd.m^−2^ with the 5-second-long stimuli.

Not all of the LCA patients demonstrated a recordable PLR to a longer stimulus (Supplementary Table S1). Three subjects (3/21 = 14%) showed no response to both the shorter (not shown) and longer stimuli (Supplementary Fig. S3). Measurements performed over at least 7.8 seconds following the stimulus onset showed no variations of pupillary diameter from baseline, ruling out existence of substantially delayed responses at this irradiance. In terms of clinical assignment of visual acuity, there was a tendency of greater ratio of subjects with LP versus NLP in the group with detectable PLR (responders: 12 LP, 6 NLP; nonresponders 1LP, 2 NLP; Supplementary Table S1). In terms of FST sensitivity losses, there was an overlap between the two groups (responders: 2.7 to >8 log; nonresponders: 7.6 to >8 log; Supplementary Table S1). Baseline pupil diameters were significantly smaller in the nonresponders (responders = 6.1 ± 1.3 mm; nonresponders = 2.9 ± 0.8 mm; *P* = 0.004). There was clear visibility of the pupil throughout the recordings (Supplementary Fig. S3, insets below), and anterior segments were mostly clear in both groups (Supplementary Table S1).

### Repeatability of the Slow PLR

**Figure 3:**
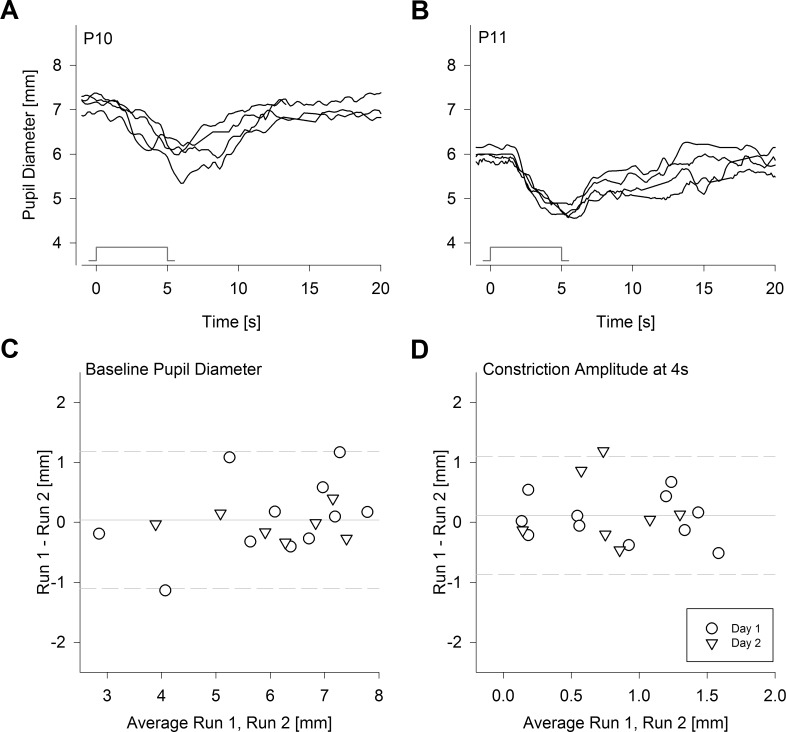
Repeatability of slow PLR in severe LCA. (**A**, **B**) PLRs recorded twice on each of 2 consecutive days in two representative patients P10 (**A**) and P11 (**B**) with the maximum achromatic stimulus of pupillometer I. Stimulus monitors show the onset and offset of the 5-second-long stimulus. (**C**, **D**) Repeatability of baseline pupil diameter (**C**) and constriction amplitude of the slow PLR at 4 seconds after stimulus onset (**D**). Limits of agreement (95%; *dashed lines*) shown for 11 patients; seven of these patients also have pairs of PLRs recorded on the second day. *Solid line* indicates mean difference.

Next, we examined the repeatability of the slow PLR in severe LCA patients. In a subset of 11 severe LCA patients who were responders, we obtained data for intravisit variation by recording a second PLR after a >1.5-minute interval following the first PLR. In 7 of these 11 patients, pairs of PLRs were repeated on a second day to obtain data for intervisit variation. As representative results from P10 and P11 demonstrate (Figs. 3A, 3B), there were some variations in baseline pupil diameters, response latencies, and amplitudes; however, the differences tended to be small, and the temporal dynamics of the PLRs tended to be consistent across two runs of two sessions performed on consecutive days. Baseline pupil diameters did not have a bias between the first and second runs (mean difference, 0.04 mm), and 95% limits of agreement were −1.1 to 1.2 mm (Fig. 3C). PLR amplitude measured at the fixed time of 4 seconds also did not have a bias between the first and second runs (mean difference 0.1 mm), and 95% limits of agreement were −0.9 to 1.1 mm (Fig. 3D). In general, the variability of the slow PLR in severe LCA was not greater than the fast PLR in less severe forms of LCA.^32^

### Limits of the Reciprocity Between Stimulus Irradiance and Duration

**Figure 4:**
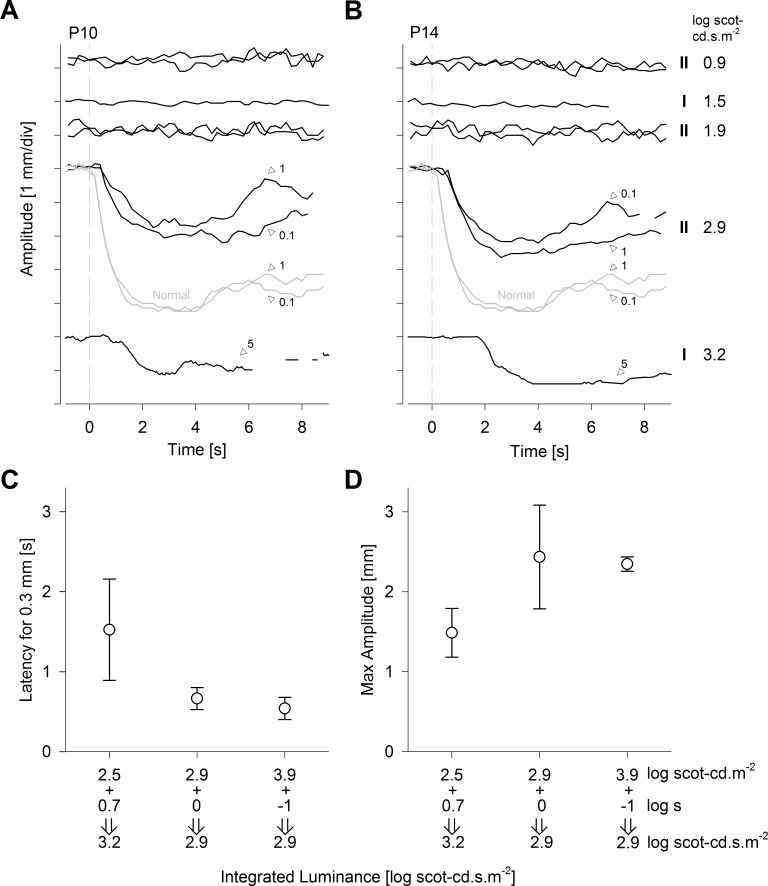
Reciprocity of stimulus luminance and duration driving slow PLR. (**A**, **B**) Pupil constriction amplitudes recorded in two LCA subjects with increasing luminous energies. The pupillometer (I or II) and the luminous energy of the stimuli are shown to the right of the traces in **B** and stimulus durations are shown on key traces. The lowest trace of P10 is interrupted with three blinks starting around 6 seconds. (**C**, **D**) The average latency and amplitude of detectable PLRs evoked by different combinations of stimulus luminance and duration. *Error bars*: ± 1 SD.

With stimulus-locked PLR, shorter latency responses allow less time for the development of confounding influences of nonphotic processes to pupil diameter changes. This is especially true in severe LCA eyes with substantial oculomotor instability. We hypothesized that PLR latencies may be accelerated with the use of higher luminance stimuli. Figure 4 presents a combination of results obtained with both the standard and higher luminance pupillometers in a subset of four patients (P7, P10, P11, and P14). Nondetectable PLRs were confirmed with 0.1-second-long stimuli at luminances of 1.9, 2.5, and 2.9 log scot-cd.m^−2^ and with 1-second long stimuli at luminances of 0.9 and 1.9 log scot-cd.m^−2^ (Figs. 4A, 4B). On the other hand, detectable PLRs were recorded with 0.1-second-long stimuli at 3.9 log scot-cd.m^−2^, and with 1-second-long stimuli at 2.9 log scot-cd.m^−2^ (Figs. 4A, 4B). These results suggested a PLR threshold occurring above 1.9 but below 2.9 log scot-cd.s.m^−2^ for a range of pupillometer II white stimuli between 0.1 and 1 second in duration. Pupillometer I results demonstrating a PLR threshold above 1.5 but below 3.2 log scot-cd.s.m^−2^ for white stimuli between 0.1 and 5 seconds in duration (Figs. 4A, 4B) were consistent also. Interestingly, 0.1- and 1-second stimulus conditions appear to show near complete temporal summation and a faster response, whereas the 5-second stimulus evoked a slower (Fig. 4C) and smaller (Fig. 4D) response, suggesting that the reciprocity between irradiance and duration may start to break down between 1 and 5 seconds in direct PLR recordings using a natural pupil. Potential contributors to this observation may include light adaptation of ipRGCs.^45^ Even though a detailed examination of the temporal tuning properties of the PLR detected in severe LCA was beyond the scope of the current work, our results provide some insight to the limits of reciprocity between stimulus irradiance and duration, and suggest an ideal stimulus duration of 1 second or shorter and a stimulus luminance of 2.9 log scot-cd.s.m^−2^ or higher, which is similar to the conclusion reached by previous investigators.^8^

## Discussion

LCA refers to a group of inherited retinopathies associated with vision loss from early life, and it is caused by more than 20 distinct monogenic defects affecting rod and cone photoreceptors or the RPE.^46−48^ Some genetic forms of LCA, such as those caused by *GUCY2D*, demonstrate near normal photoreceptor structure.^30,31,49^ Other forms, such as those caused by *CEP290* or *NPHP5*, show retained central cone photoreceptor,^24,26,29,30,32,50,51^ and yet others, such as *RPGRIP1* and *AIPL1*, are associated with widespread outer retinal degeneration.^25,27,30^ Inner retinal structures, such as retinal ganglion cells and the nerve fiber layer, are often retained on OCT^49,52^ and on histology.^53,54^

Visual function in LCA shows a wider spectrum; there is often some remaining vision, and perceptual tests (such as visual acuity) provide quantifiable but subjective evidence of a functioning retinogeniculostriate pathway. Objective approaches to evaluate the fidelity of postretinal transmission of light-evoked signals include functional magnetic resonance imaging^52,55^ and pupillometry.^27,28,31–37^ Diagnostic signs of LCA are often described as including “sluggish pupils,” but most LCA subjects with some retained vision demonstrate rapid PLRs that are not substantially different from normal PLRs, especially when high-luminance stimuli are used.^8,23,27,28,31–37^ A subset of LCA patients have either no perception of lights or they perceive only bright lights but have no form vision or motion perception: visual acuity is clinically assigned as NLP or LP,^56^ and quantitative FST measures show extremely elevated or unmeasurable thresholds.^27,28,31,41^ Pupillary responses of these severe LCA patients were the subject of the current investigation.

### Slow PLR in Severe LCA

The majority of the patients with severe LCA showed a detectable PLR in response to a bright stimulus with long duration. The resulting stereotypical PLR was very insensitive to light (threshold elevations of >8 log units at early times and ∼4 log units at late times), with a slow latency, small amplitude, and a steep stimulus–response curve. The slow PLR appeared to be independent of the structure and topography of the outer retina and included patients with retina-wide (*GUCY2D*), macular (*CEP290* and *NPHP5*) or foveal (*RPGRIP1*) retention, as well as those with retina-wide degeneration (*AIPL1*-associated LCA). A similar stereotypical PLR was also recorded in one case of syndromic Batten disease due to *CLN3* mutations involving severe retina-wide degeneration of the outer retina. To our knowledge, the literature to date contains pupillometry results from four comparable patients. One subject was an 87-year-old affected female with autosomal dominant cone-rod dystrophy and NLP.^21^ The temporal profile and amplitude of the PLRs were not provided, but the pupils were only responsive to bright lights with a threshold near 14.5 log photons.cm^−2^.s^−1^ at a peak sensitivity of 481 nm with 10-second duration stimuli. Another case was a 58-year-old male with retinitis pigmentosa and NLP who showed slow pupillary responses with a threshold near 13 log photons.cm^−2^.s^−1^ at 480 nm with a 6-second duration stimulus.^22^ A third case was a 41-year-old female with LCA (genotype and retinal structure not reported) and LP vision demonstrating a delayed PLR to a bright blue stimulus of 2.6 log phot-cd.m^−2^ luminance and 1-second duration.^8^ And the fourth case was a 36-year-old female with *CEP290*-associated LCA and LP vision who also showed a delayed PLR to a bright blue stimulus of 2.6 log phot-cd.m^−2^ luminance and 1-second duration.^23^ Our PLR results in 18 severe LCA patients recorded with 2.4 log phot-cd.m^−2^ (∼13 log quanta.cm^−2^.s^−1^) achromatic stimuli of 5-second duration appear to be generally similar to those recorded in the four patients previously reported in terms of the elevated response thresholds, small amplitudes, and delayed latencies. However, the threshold of slow PLR in severe LCA appears to be at least 1 log unit higher than the commonly accepted threshold of intrinsic light responses of ipRGCs.^4,7,39^

### What Is the Physiological Origin of the Slow PLR in Severe LCA?

The initial rapid constriction phase of the normal human PLR that quickly follows the onset of a bright long-duration stimulus is thought to be driven mostly by the photoreceptors of the outer retina, whereas the intrinsic signaling by the ipRGCs of the inner retina is thought to contribute strongly to the late sustained constriction after stimulus offset.^4,10,12,15^ The full time course of the human PLR resulting from activation of only ipRGCs without outer retinal input is not well known, but primate and murine results suggest a slower constriction with a smaller amplitude and substantially reduced light sensitivity that peaks near 480 nm.^3,7,10^ Therefore, parsimony dictates that the slow PLRs described in the current work in severe LCA are likely dominated by the activation of the melanopic circuit in isolation from outer retinal input, consistent with previous interpretations of the origins of PLR in patients with severe vision loss.^8,21–23^ Measurement of spectral sensitivity of the slow PLR in well-characterized patients with severe LCA may help further support this interpretation in the future. Assuming the PLR recorded in severe LCA to represent the isolation of the melanopsin-driven component of the normal PLR, our results support the notion of PIPR^4,10,16–20^ as a surrogate marker for melanopic function. However, the exact time interval utilized to evaluate the PIPR must be carefully considered in relation to the stimulus used. For our stimulus and recording conditions, the putative melanopic PLR in LCA did not overlap with the normal response on average until approximately 10 seconds poststimulus offset (Fig. 2C).

Perhaps surprisingly, a PLR was not detectable in some severe LCA patients (Supplementary Fig. S3) with the long-duration maximal luminance of pupillometer I. The simple interpretation of such a result would be that some patients are lacking melanopic signaling due to degenerate ipRGCs, undiagnosed mechanical pupil defects, or abnormalities in the afferent and/or efferent component of the retino-pretectal tract. There are several other likely possibilities also. In one of the nonresponders (P16), pupils did not dilate after a 40-minute period of dark adaptation. Small pupils in the dark make detection of light-dependent PLRs challenging or impossible, both due to decreased retinal irradiance as well as limited range of constrictions. The reasons for nondilation in P16 are not known but could include chronic signaling from diseased photoreceptors^57^ as well as other nonretinal causes. In the two remaining nonresponders (P3 and P5), the pupil was dilated enough to allow for detection of a PLR, but none was detected. In these patients, it may be that the slow PLR thresholds were elevated just above the maximal luminance afforded by pupillometer I, possibly due to a coincidental melanopsin gene polymorphism.^58^ Future studies using pupillometer II with ∼1.3 log unit greater luminance will test this hypothesis.

### PLR as an Inclusion Criterion and Objective Outcome Measure in Treatment Trials

Direct PLR evoked with short-duration stimuli presented to dark-adapted eyes has been used in human clinical trials of gene therapy in the *RPE65* form of LCA^35,37^; additionally, a more complex PLR evoked with stimuli alternating between the eyes has also been used.^59^ Importantly, *RPE65*-LCA is one of the least severe forms of LCA with no reports of patients with congenital lack of light perception.^60^ It is not surprising, therefore, that there were outer retinal photoreceptor-driven transient PLRs in all *RPE65*-LCA patients before treatment and perceptual improvements resulting from gene therapy were associated with improvements of PLR sensitivity.^35,37^ Patients with severe LCA with no outer retinal photoreceptor function undergoing future treatment trials will be much more challenging than *RPE65*-LCA patients. Potential treatments of photoreceptor replacement for these patients include gene augmentation therapy, optogenetics, stem cells, and electronic chip implants.^61^ A prerequisite to all of these treatment pathways is the demonstration of the existence of a functioning transmission pathway that can carry improved light-evoked signals from the retina to the brain.

We hypothesized that melanopic PLR function can be used to demonstrate the fidelity of the retino-pretectal tract in severe LCA patients with congenital and complete blindness even when outer retinal function is lacking. Surprisingly, however, LCA patients did not show detectable PLRs to bright and short-duration stimuli expected to be well above ipRGC thresholds (Fig. 1). We then used a longer-duration stimulus to take advantage of the temporal integration properties of the ipRGCs consistent with previous work.^3,44,62^ Most patients showed a detectable but slow PLR likely to represent the melanopic function in isolation (Fig. 2). However, with direct PLR recording methods, where the stimulus is presented to the same eye as the measured PLR, stimulus durations longer than the latency of the pupil constriction would result in complex temporal changes to the retinal irradiance. This complexity can be bypassed with consensual PLR recording methods where the stimulus is presented to a dilated eye and the pupil response is measured in the contralateral undilated eye.^4,10,12,16,63^ The consensual approach would not be practical as an outcome for LCA treatment in which each eye needs to be evaluated independently. Here we present preliminary data (Fig. 4) that support the use of short-duration high-luminance stimuli in direct PLR recording methods to activate melanopic PLRs independently in each eye. The resulting PLR showed a much faster activation compared to the long-duration stimulus, though it was still slower than the normal response. A technique such as described with the pupillometer II that produces detectable PLRs in the full spectrum of LCA would provide not only evidence of postretinal transmission of light-evoked signals but also would be useful as an objective outcome of treatment effects related to safety as well as efficacy.

## Supplementary Material

Supplement 1
