# Supplementary material for: Pupillary Light Reflexes in Severe Photoreceptor Blindness Isolate the Melanopic Component of Intrinsically Photosensitive Retinal Ganglion Cells
Source: Invest Ophthalmol Vis Sci. 2017 Jul;58(7):3215–24. doi: 10.1167/iovs.17-21909 (PMC5490362; doi:10.1167/iovs.17-21909)

Pupillary Light Reflexes in Severe Congenital Photoreceptor Blindness Isolate the Melanopic Component of Intrinsically-photosensitive Retinal Ganglion Cells

Jason Charng, Samuel G Jacobson, Elise Heon, Alejandro J. Roman, David B. McGuigan 3rd, Rebecca Sheplock, Mychajlo S. Kosyk, Malgorzata Swider, Artur V. Cideciyan

SUPPLEMENTARY MATERIALS

SUPPLEMENTARY TABLE 1
SUPPLEMENTARY FIGURE LEGENDS AND FIGURES FOR:
- FIGURE S1
- FIGURE S2
- FIGURE S3

Suppl. Table 1. Clinical and molecular characteristics of LCA patients

Patient	Age(yrs) /gender	Allele 1	Allele 2	Eye	VA	Lens	FST *(log)*	PLR to 5s
GUCY2D								
P1	3/M	c.91_92 insC	Glu103Lys	RE	NLP	Clear	N/A	Y
P2	9/M	Arg768Trp	Arg768Trp	LE	NLP	Clear	>8	Y
P3	11/M	Arg768Trp	Ser981 del1bp	LE	NLP	2^{+} PSC	>8	N
P4	14/F	Gln156x	Ser819 del1bp	RE	LP	Clear	2.7	Y
P5	26/M	Ser981 del1bp	Ser981 del1bp	RE	LP	Clear	7.6	N
P6	37/F	Leu865 del1bp	Met476 ins10bp	LE	LP	Clear	>8	Y
CEP290								
P7	13/F	c.2991+1655A>G	c.1189+1G>C	RE	NLP	Clear	>8	Y
P8	9/M	c.2991+1655A>G	c.289G>T	LE	NLP	1^{+} PSC	>8	Y
P9	13/M	c.2991+1655A>G	c.4115_4116delTA	RE	LP	Clear	7.2	Y
P10	14/F	c.2991+1655A>G	c.547_550delTACC	RE	LP	Clear	7.1	Y
P11	17/F	c.2991+1655A>G	c.1550delT	LE	LP	PCS	6.9	Y
P12	18/M	c.2991+1655A>G	c.2506_2507delGA	RE	LP	Clear	7.3	Y
P13	24/F	c.2991+1655A>G	c.5491delA	RE	NLP	Clear	>8	Y
P14	29/F	c.2991+1655A>G	c.2052+1_2052+2delGT	LE	LP	Clear	7.2	Y
P15	48/F	c.2991+1655A>G	c.2594_2595delTC	RE	LP	1^{+} NS	6.6	Y
P16	51/M	c.2991+1655A>G	c.2390delA	LE	NLP	1^{+} PSC	>8	N
NPHP5								
P17	15/M	Arg489X	His506 del2cagCA	RE	LP	Clear	7.7	Y
AIPL1								
P18	23/F	Thr265Cys	Thr214Cys	LE	LP	PCS	7.1	Y
P19	40/F	Trp278X	Leu241 del3ctGAA	RE	LP	1^{+} NS	7.9	Y
RPGRIP1								
P20	14/M	c.3749-1G>T	Gln361fs	LE	LP	Clear	N/A	Y
CLN3								
P21	16/M	1.02 kb genomic del	1.02 kb genomic del	RE	NLP	Clear	>8	Y

FST, full-field sensitivity test; LE, left eye; LP, light perception; ND, not detectable; N/A, not available; NLP, no light perception; NS, nuclear sclerosis; PCS, peripheral cortical spokes; PSC, posterior subcapsular opacification; RE, right eye

SUPPLEMENTARY FIGURE LEGENDS FOLLOWED BY FIGURES

Supplementary Figure 1: Maximal pupil diameter measurements at various gazes. (**A**) In a normal dark adapted eye, iris images are shown at central as well as eccentric gaze positions. Yellow outlines demarcate the edges of the pupil in each image. Pairs of arrows show the maximal pupil estimate for each eccentric gaze. In the central view (Image 5), a pair of horizontal and a pair of vertical arrows are used to indicate that both measurements are equal as the pupil is circular in this view. (**B**) Maximal pupil diameters estimated at various gaze directions, the numbers on the x-axis correspond to the gaze directions marked in Panel A. Horizontal gray line indicates pupil diameter estimated in the central position (Image 5).

Supplementary Figure 2: Spectral characteristics of white stimuli for Pupillometer I (A) and II (**B**).

Supplementary Figure 3: No detectable PLR in a subset of severe LCA. Three subjects (P3, P5, P16) showing no discernible PLR to the 5s stimulus with Pupillometer I. Images below the x-axis indicate clear pupil visualization throughout the recording. Stimulus monitors are shown.

Suppl. Figure 1

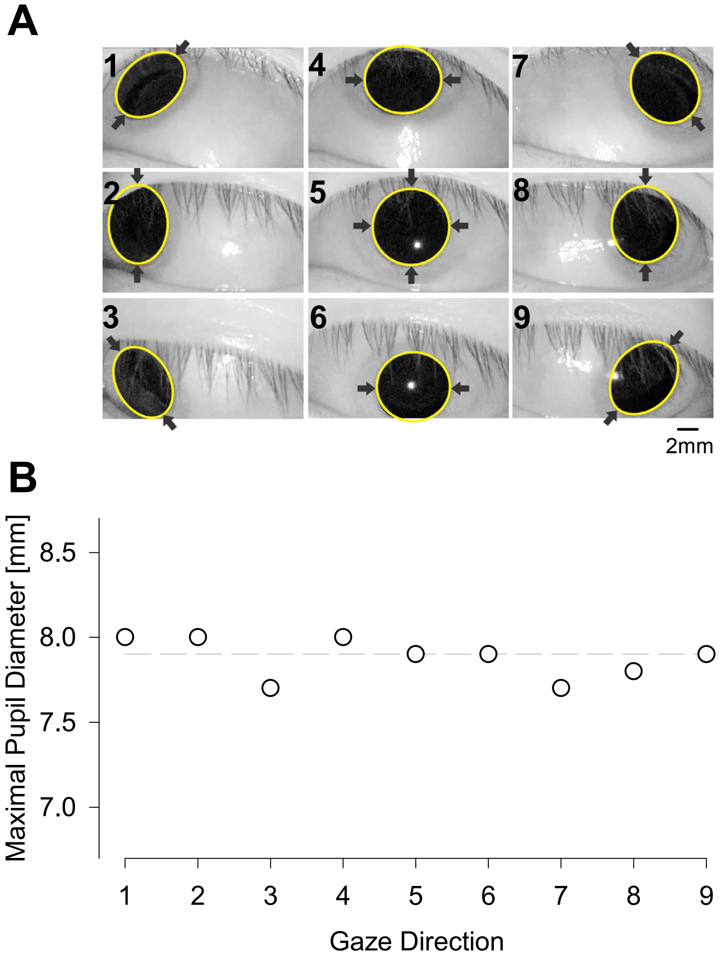

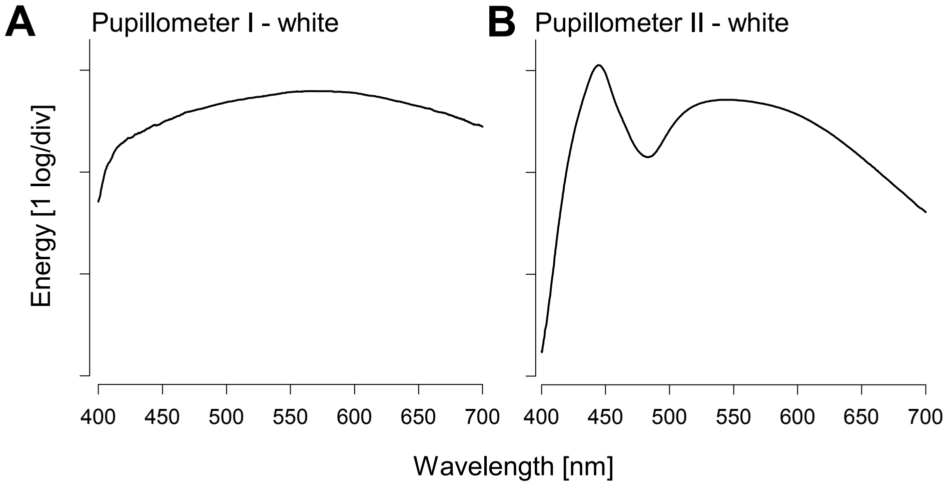
A
Pupillometer I - white
B
Pupillometer II - white
Energy [1 log/div]
400
450
500
550
600
650
700
Wavelength [nm]

Suppl. Figure 3

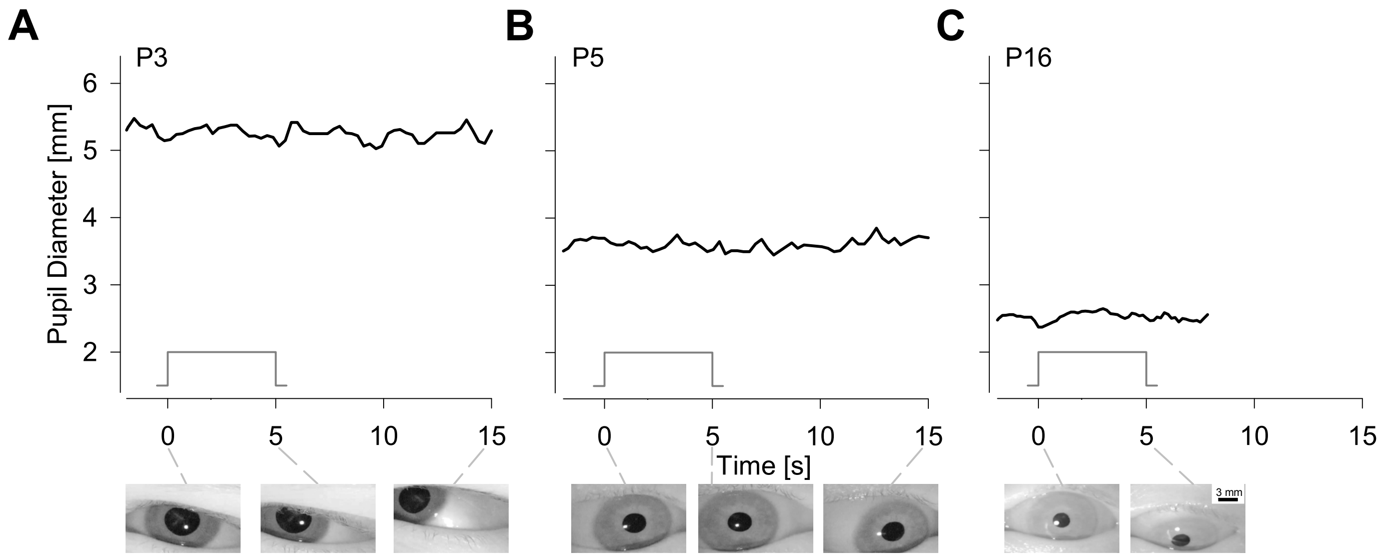

Supplement: Supplement 1 [file iovs-58-07-16_s01.pdf]
